# Immune Response to Vaccination against COVID-19 at Different Second-Dose Intervals and Their Associations with Metabolic Parameters

**DOI:** 10.3390/vaccines11010149

**Published:** 2023-01-10

**Authors:** Łukasz Szczerbiński, Michał Andrzej Okruszko, Maciej Szabłowski, Jędrzej Warpechowski, Adam Paszko, Anna Citko, Paulina Konopka, Witold Bauer, Adam Jacek Krętowski

**Affiliations:** 1 Clinical Research Centre, Medical University of Bialystok, Sklodowskiej-Curie 24A, 15-276 Bialystok, Poland; 2 Department of Endocrinology, Diabetology and Internal Diseases, Medical University of Bialystok, Sklodowskiej-Curie 24A, 15-276 Bialystok, Poland; 3 Center for Genomic Medicine, Massachusetts General Hospital, 185 Cambridge Street, Boston, MA 02114, USA; 4 Programs in Metabolism and Medical and Population Genetics, Broad Institute of MIT and Harvard, 75 Ames Street, Cambridge, MA 02142, USA

**Keywords:** COVID-19 vaccines, antibodies, humoral response, SARS-CoV-2, obesity, metabolic parameters

## Abstract

Obesity and diabetes are associated with severe outcomes of coronavirus disease (COVID-19). Vaccines against severe acute respiratory syndrome coronavirus 2 (SARS-CoV-2) have been proven protective against infection and severe COVID-19. However, the immune response of metabolically burdened individuals to the vaccines remains unclear. Thus, we aimed to assess whether the metabolic status of individuals affects their humoral immune responses to the vaccination. Moreover, we evaluated whether the interval between the first two doses influenced antibody concentration. Sixty-seven individuals (21 males, 46 females) were vaccinated with the BNT162b2 mRNA COVID-19 vaccine. Fifty-four individuals were vaccinated with the second dose after 3 weeks and 13 after 5 weeks. We measured the antibody titers in all participants during the 19-week follow-up period. Patients diagnosed with COVID-19 were excluded. In the 5-week interval group, a significantly higher level of maximal antibody titers was observed. However, there were no differences in antibody concentrations after 19 weeks and no significant correlation between cardiometabolic factors and humoral response. The elongation of second-dose timing to 5 weeks leads to a higher acute antibody response but does not change long-term levels of antibody titers. Moreover, dysregulation of metabolic parameters does not lead to a diminished immune response to vaccination.

## 1. Introduction

Coronavirus disease 2019 (COVID-19), caused by severe acute respiratory syndrome coronavirus 2 (SARS-CoV-2), is one of the most far-reaching epidemics in the modern world. This pandemic has changed the lives of most people and killed nearly 14.9 million globally, according to World Health Organization (WHO) reports [1]. The primary situation has changed drastically due to the rapid, record-breaking development of vaccines against COVID-19, which have significantly reduced mortality and the severity of the disease [2,3,4,5,6,7,8]. Due to the short production time and low-level biosafety requirements, mRNA vaccines remain the most quickly accessible vaccines during the global pandemic [9]. In these vaccines, mRNA encodes the spike (S) protein, resulting in anti-spike antibody (anti-S-ab) synthesis, whereas infection results in the production of anti-nucleocapsid (anti-N-ab) antibodies. This enables differentiation if the tested individual is infected with SARS-CoV-2 [10]. Current vaccination protocols for mRNA vaccines require at least two doses in a relatively short time (usually 3 weeks) to achieve basic immune protection and boosters to maintain immunity for longer periods. However, the effect of the interval between the first two doses on the long-term immune response remains unclear.

Now, it is well known that the main determinants of the course and clinical outcome of the COVID-19 disease are elderly age, obesity, and metabolic dysregulation, such as diabetes, dyslipidemia, and hypertension [11]. However, the role of these factors in the immune response to the vaccination remains unclear. Thus, our study aimed to evaluate whether the interval between the first two doses of the vaccine and metabolic parameters affected the humoral response to the COVID-19 vaccine.

## 2. Materials and Methods

### 2.1. Study Group

This study was conducted at the Clinical Research Center of the Medical University of Bialystok in Bialystok, Poland. In the analysis, we included 67 (21 male, 46 female) participants aged between 18 and 65 years without any contraindications for vaccination and who were not diagnosed with COVID-19 before or during study participation. The participants were divided into two groups depending on the timing of the second dose of the vaccine: 3 (n = 54) or 5 (n = 13) weeks. Our study was conducted as a part of the national vaccination program. All the participants from the 3-week interval group were vaccinated in the “classic” three-week interval manner. However, there was a time in Poland when vaccine availability was temporarily limited. Thus, the national recommendations allowed the extension of the interval to 5 weeks. However, that was the case only for a short period of time, and when the availability of the vaccine increased, the recommendations canceled the extension to the 5-week rule. During that time, we could include only a small number of subjects who started the vaccination scheme at a 5-week interval. The concentration of antibodies and assessment of clinical parameters were tested before receiving the first dose of the vaccine (visit 1), then checked every week for 7 weeks (visits 2–8), followed by every 4 weeks (visits 9–11). The follow-up period was 19 weeks. Diagnosis of COVID-19 was defined as obtaining a positive polymerase chain reaction (PCR) or antigen test result or detection of antibodies against nucleocapsid protein (anti-N-ab).

All patients provided written informed consent to participate in the study. The study was conducted in accordance with the Declaration of Helsinki and adhered to good clinical practice guidelines. The study protocol was approved by the Bioethics Committee of the Medical University of Bialystok (approval number: APK.002.79.2021).

### 2.2. Blood Sampling

Samples were collected from venous blood using S-Monovette (Sarstedt, Germany) blood collection tubes (K3 EDTA and Serum). Serum tubes were centrifuged immediately after collection (1 h at 10,000 RPM for 10 min), and the collected serum was used for antibody and biochemical measurements.

### 2.3. Antibodies, Biochemical, Anthropometric, and Body Composition Measurements

Anti-S-ab, total vitamin D, anti-thyroid peroxidase antibodies (aTPO), interleukin 6 (IL-6), ferritin, thyroid-stimulating hormone (TSH), free triiodothyronine (fT3), and free thyroxine (fT4) were measured using a double-antigen sandwich electrochemiluminescence immunoassay (Cobas e411, Roche Diagnostics, Switzerland), which utilizes microparticles coated with streptavidin to isolate unbound substances before applying a voltage to the electrode. The measuring range of Cobas e411 is between 0.40 and 250 U/mL; a concentration of <80 U/mL is regarded as negative, whereas ≥80 U/mL is regarded as positive.

Biochemical measurements of serum triglyceride (TG), total cholesterol (TChol), high-density lipoprotein cholesterol (HDL), creatinine, aspartate aminotransferase (AST), alanine aminotransferase (ALT), low-density lipoprotein cholesterol (LDL), homocysteine, and C-reactive protein (CRP) concentrations were determined using colorimetric methods with a Cobas c111 automatic chemistry analyzer (Roche Diagnostics, Switzerland).

Haemoglobin A1c (HbA1c) was measured by the high-performance liquid chromatography (HPLC) method on Bio-Rad D-10 (Bio-Rad Laboratories, Hercules, CA, USA), which is certified by the National Glycohemoglobin Standardization Program and International Federation of Clinical Chemistry and Laboratory Medicine and has a measuring range between 18 and 179 mmol/mol. Whole blood samples were diluted and injected into the analytical cartridge automatically by the device. Subsequently, a buffer gradient of progressive ionic strength was utilized to separate miscellaneous fractions of hemoglobin, which were later measured using 415 nm spectrophotometry.

Anthropometric measurements and body composition analyses were performed using a calibrated stadiometer (SECA 264; SECA, Hamburg, Germany) and an electronic scale (SECA 769; SECA, Hamburg, Germany). Body mass index (BMI) was calculated as body mass (kg) divided by height (m) squared. Body composition assays were performed with a bioimpedance test using InBody 720 (InBody, Cerritos, CA, USA). The total skeletal muscle mass (SMM), body fat mass (FM), percent body fat (fat%), and visceral fat area (VAT) were measured.

### 2.4. Statistics

Continuous data were grouped by the length of the interval between the first and second doses of the BNT162b2 mRNA COVID-19 vaccine and summarized using descriptive statistics (Table 1 and Supplementary Table S1). The normality of data distribution was tested using the Shapiro–Wilk test. We used the two-tailed unpaired t-test to compare normally distributed data and the two-tailed Mann–Whitney U test for non-normally distributed data. The difference in sex structure between the groups was checked using the chi-squared test. Spearman’s rank correlation coefficient was used to assess the relationship between clinical variables and antibody responses. Statistical significance was set as <0.05. R software [12] was used to perform the analyses and plot the figures.

## 3. Results

### 3.1. Clinical Characteristics of Studied Groups

There were no significant differences between the groups in sex distribution. The 5-week interval group was significantly older and presented a more unfavorable profile for several metabolic parameters, including BMI, body composition, systolic blood pressure, and triglycerides. However, we detected no differences in HbA1c, cholesterol (total, HDL, and LDL), thyroid status (TSH, fT3, and fT4), liver aminotransferases (AST and ALT), or inflammation parameters (CRP and IL-6), as the *p*-value was greater than 0.05, indicating a lack of differences between the two groups. The detailed clinical characteristics of both groups are shown in Table 1.

### 3.2. Comparison of Antibody Response to Vaccination between Groups

**Figure 1:**
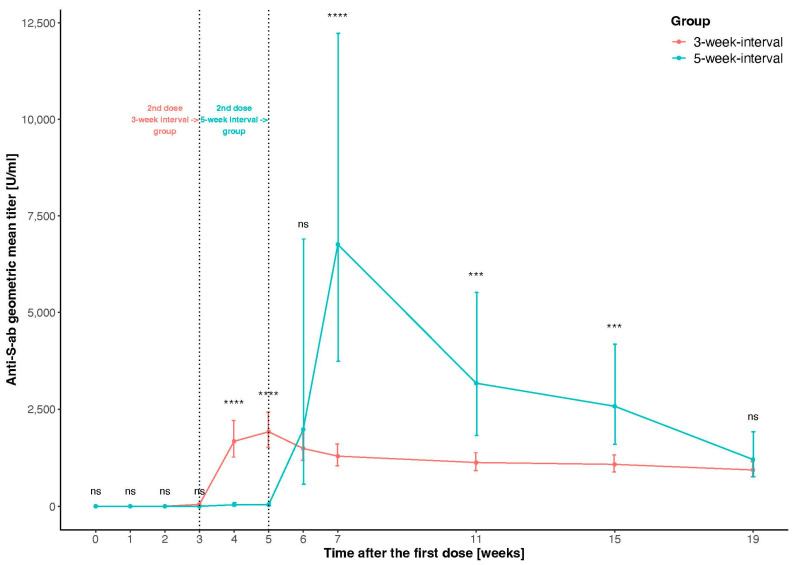
Response in anti-s-ab concentrations to COVID-19 vaccine in 19-week observation. Presented are the standardized geometric means and the 95% confidence intervals of anti-S-ab concentrations. Significance levels: ns *p* ≥ 0.05; ***p < 0.001; **** *p* < 0.0001.

In both groups, the maximal anti-S-ab titer level was observed approximately 2 weeks after the second dose of the vaccine. The maximal antibody concentration was significantly higher in the 5-week interval group than in the 3-week interval group (*p* < 0.001). The observed difference was almost four times higher when the second dose was administered after a longer period (geometric mean maximal titer: 1916.53 U/mL vs. 6762.20 U/mL in the 3- and 5-week interval groups, respectively). Interestingly, we found no difference in antibody concentrations between groups on the last follow-up visit, 19 weeks after the first vaccine dose (*p* = 0.202) (geometric mean titer at the last visit: 934.07 U/mL and 1203.70 U/mL in 3- and 5-week interval groups, respectively) (Figure 1, Supplementary Table S1).

### 3.3. Clinical Parameters and Antibody Response to the Vaccine

**Figure 2:**
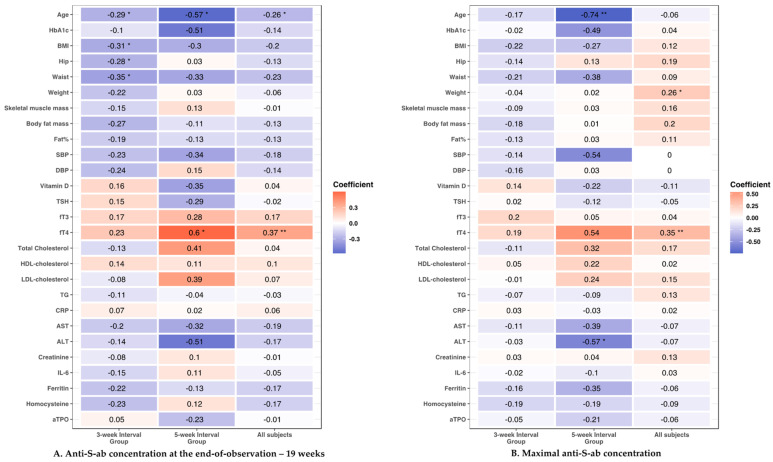
Correlations between clinical parameters and antibody responses (**A**) anti-S-ab concentration at the end-of-observation—19 weeks; (**B**) maximal anti-S-ab concentration). Significance levels: * *p* < 0.05; ** *p* < 0.01; Abbreviations: HBA1c—Glycated A1c Fraction of Hemoglobin; BMI—Body Mass Index; Hip—Hip Circumference; Waist—Waist Circumference; SBP—Systolic Blood Pressure; DBP—Diastolic Blood Pressure; TSH—Thyroid Stimulating Hormone; fT3—Free Triiodothyronine; fT4—Free Thyroxine; HDL—High Density Lipoprotein Cholesterol; LDL—Low Density Lipoprotein Cholesterol; TG—Triglycerides; CRP—C-Reactive Protein; AST—Aspartate Aminotransferase; ALT—Alanine Aminotransferase; IL-6—Interleukin-6; Fat%—Body Fat Percentage; aTPO—Anti-Thyroid Peroxidase Antibodies.

We analyzed whether the antibody responses to vaccination, both maximal and end-of-follow-up (19 weeks), correlated with age, anthropometric, and cardiometabolic parameters. The analysis was performed in each group separately and for all studied participants. The results of the analysis are shown in Figure 2.

#### 3.3.1. Age

We found significant negative correlations between age and end-observation anti-S-ab titer level in both groups separately and in all studied participants analyzed together (5-week interval group: r = −0.57, *p* < 0.05; 3-week interval group: r = −0.29, *p* < 0.05; all participants: r = −0.26, *p* < 0.05). Moreover, in the 5-week interval group, we found significant negative correlations between age and maximal anti-S-ab titer level (r = −0.74; *p* < 0.01).

#### 3.3.2. Anthropometric Parameters

We observed several significant correlations between anthropometric measurements of the participants and their humoral responses. Negative correlations were observed between the end-observation anti-S-ab titer level and hip and waist circumferences in the 3-week interval group (hip circumference: r = −0.28, *p* < 0.05; waist circumference: r = −0.35, *p* < 0.05). Similar results were observed for BMI (r = −0.31, *p* < 0.05). However, these correlations were not significant when analyzing the 5-week interval group and all participants. We did not identify any significant correlations between antibody responses and weight, fat content, visceral fat mass, or skeletal muscle mass.

#### 3.3.3. Cardiometabolic Parameters

Interestingly, among all cardiometabolic parameters studied, the only significant correlations found were between antibody response and fT4 and ALT. We found that the serum level of fT4 correlated positively with the end-observation anti-S-ab titer level in the 5-week interval group (r = 0.6, *p* < 0.05) as well as among all participants (r = 0.37, *p* < 0.01), but not in the 3-week interval group (r = 0.23, *p* = 0.09). Moreover, fT4 was positively correlated with the maximal anti-S-ab titer level in all participants (maximal titer: r = 0.35, *p* < 0.01). ALT serum levels were negatively correlated with maximal anti-S-ab titers in participants in the 5-week interval group (r = −0.57, *p* < 0.05). We did not observe any statistically significant correlations between the humoral response, either maximal or after 19 weeks of follow-up, and the remaining measured cardiometabolic parameters (blood pressure, HbA1c, lipids, AST, creatinine, Vitamin D3, or IL-6).

## 4. Discussion

Our results showed that the extended time between the first and second doses of BNT162b2 mRNA vaccine led to a higher maximal response in anti-S-antibody production. This is consistent with previous studies [13,14,15,16]. However, our results show that this effect is only temporary, as 19 weeks after the first dose, the concentration of anti-S-antibodies is not dependent on the duration of the interval between the doses. Delay of the second dose of the vaccine appears to result in almost four times higher maximal concentration of antibodies. However, it is still unknown whether the peak level of antibodies has clinical relevance. It would be valuable to evaluate whether the maximal concentration of antibodies is related to better cellular responses and protection against viruses. In our study, we did not assess the cell-mediated immune responses. However, it is suggested that it does not differ significantly in prolonged interval dosing schemes, and a higher neutralizing antibody titer level does not correlate with an improved cellular immune response [17]. Despite many ongoing attempts, the protective antibody titer level has not yet been precisely determined. More large-cohort studies are required [18]. These facts should be considered when administering standard- or prolonged-interval vaccination schemes.

Results of our study suggest negative correlations between BMI, hip circumference, waist circumference, and antibody response, which reflects the findings of other studies [19,20,21]. There is a link between obesity and impaired seroconversion following the administration of vaccines against different diseases, as well as an increased likelihood of infection, even among obese individuals with good-functioning seroconversion [22,23]. This result could be explained by the fact that a higher adipose tissue content (the main driver of increased BMI) leads to a disproportion of adipocytokines, causing low-grade chronic inflammation, which finally stimulates the immunosenescence of B cells and impinges on the production of antibodies [24,25]. However, a handful of studies have suggested no association between BMI and humoral response following COVID-19 vaccine administration [19,26,27,28]. In our study, we found a significant correlation between the aforementioned parameters in the 3-week interval group but not in the 5-week interval group. It is important to note that participants in the 3-week interval group had significantly lower BMI and better general metabolic status than those in the 5-week interval group. This suggests the effect of adiposity might be relevant only in non-obese patients. However, this hypothesis requires further investigation.

Another finding of our study was the negative impact of age on antibody responses in our participants. Similar results were reported in other studies [19,29,30]. A possible explanation is that aging negatively influences innate and adaptive immune responses [31]. Moreover, different qualities of memory B cells and plasma compartments, including recombination of the class switch and differentiation into plasma cells, have already been observed in older people [32].

In the present study, we also evaluated the impact of homocysteine and ferritin levels on antibody response to vaccination. Some studies suggest that Hcy can be a valuable biomarker for the risk of severe COVID-19 [33,34,35]. To the best of our knowledge, there is no scientifically proven data reporting the level of homocysteine and humoral response following vaccine administration. The same conclusion can be drawn for ferritin, which is also associated with poor COVID-19 prognosis, but there are no studies regarding ferritin as a biomarker of humoral response among vaccinated individuals [36]. In our study, we showed that, despite their possible engagement in COVID-19 pathophysiology and disease risk, homocysteine and ferritin do not seem to influence response to the COVID-19 vaccine in terms of antibody titers.

Finally, we found an interesting positive correlation between the levels of fT4 and anti-S-ab titers in our participants. There are reports of possible thyroid complications of COVID-19, where a decrease in fT3 and TSH concentrations and cases of subacute thyroiditis have been observed [37,38]. Moreover, several cases of thyroid dysfunction following COVID-19 vaccination have been described [39,40,41]. Initial studies have shown that patients with autoimmune thyroiditis do not present impaired immunological responses to the COVID-19 mRNA vaccine [42]. In our study, we did not find significant correlations between TSH, fT3, or aTPO concentrations and antibody response. However, the positive correlation between fT4 and total (19-week) and peak responses in antibody concentrations suggests thyroid hormones may affect the immune response to vaccination. This hypothesis requires further investigation, especially in larger cohorts with a wider variety of thyroid hormone concentrations (including participants with hypo- and hyperthyroidism, which was not the case in our study).

The second dose of the COVID-19 vaccine is highly recommended in cases of low protection levels following the first dose [43]. However, in the initial phase of COVID-19 vaccination, the vaccine supply was limited, leading to the necessity of vaccine prioritization between older individuals with increased mortality and young, active people, which would decrease the possibility of virus spread [44]. The delay of the second dose of the vaccine was a possible solution, as it would increase the number of partially vaccinated individuals, but it also resulted in similar controversy. As the increased number of partially vaccinated individuals is likely to accelerate the spread of vaccine-adapted variants of the novel coronavirus, it could minimize mortality among the most vulnerable social groups. [43].

One of the limitations of our study was the relatively small sample size. Moreover, the clinical characteristics of the studied groups show that participants in the 3-week interval group are more “metabolically healthy” than those in the 5-week interval group. This could have affected the observed differences in the outcomes. However, in the interpretation of our results, and especially in assessing how phenotypic features affect antibody responses, we tried to distinguish these differences by evaluating the results of separate analyses for each group and one combining all participants. Finally, our study only evaluated the humoral response to the COVID-19 vaccine. Further studies including cellular responses should be conducted to assess the full immune response.

## 5. Conclusions

In summary, our results demonstrate that administering the second dose of the BNT162b2 mRNA vaccine after 5 weeks increases the peak titer of antibodies, which may lead to increased immunity against COVID-19 during the first weeks after vaccination (compared to standard administration after 3 weeks). However, the timing of the second dose of the vaccine did not affect the long-term (19-week) response in antibody concentrations. Another important conclusion of our study is that age, BMI, waist and hip circumferences, and thyroxine concentration can affect antibody responses to the vaccine. In contrast, homocysteine and ferritin, despite being associated with poor COVID-19 prognosis, do not affect the immune response. Total and peak antibody responses seem to be associated with fT4 concentration. We strongly believe that our research will contribute to a better understanding of the importance of the interval between doses. We also hope that this will be another step in the development of the most appropriate vaccination schedules for groups with specific metabolic conditions.

## Figures and Tables

**Table 1:** Clinical characteristics of the studied groups. The significant *p*-values (<0.05) are italicized. Abbreviations: HBA1c—Glycated A1c fraction of hemoglobin; BMI—Body Mass Index; Fat%—body fat percentage; SBP—Systolic Blood Pressure; DBP—Diastolic Blood Pressure; HR—Heart Rate; TSH—Thyroid Stimulating Hormone concentration; fT3—Free Triiodothyronine concentration; fT4—Free Thyroxine concentration; HDL-cholesterol—High-Density Lipoprotein Cholesterol concentration; LDL-cholesterol—Low-Density Lipoprotein Cholesterol concentration; TG—Triglycerides concentration; CRP—C-Reactive Protein concentration; AST—Aspartate Aminotransferase concentration; ALT—Alanine Aminotransferase concentration; IL-6—Interleukin-6 concentration.

	3-Week Interval Group		5-Week Interval Group		*p*-Value
Parameters:	Mean	sd (+/−)	Mean	sd (+/−)	
Age (years)	35.94	13.21	50.62	16.07	0.003
HbA1c (%)	5.33	0.62	6.18	1.11	0.013
BMI (kg/m^2^)	25.38	4.52	30.85	5.27	0.001
Weight (kg)	71.82	15.32	89.82	13.52	0.001
Skeletal muscle mass (kg)	27.52	6.30	32.40	7.48	0.020
Body fat mass (kg)	21.90	8.86	31.79	9.96	0.005
Fat%	29.88	9.18	35.34	9.78	0.085
SBP (mmHg)	126.02	19.64	141.38	17.24	0.011
DBP (mmHg)	82.16	13.05	84.15	11.62	0.600
Vitamin D (ng/mL)	28.99	12.94	24.26	9.82	0.160
TSH (uIU/mL)	1.68	0.68	1.69	1.43	0.580
fT3 (pg/mL)	3.51	0.48	3.44	0.38	0.760
fT4 (ng/dL)	1.30	0.15	1.33	0.29	0.670
Total cholesterol (mg/dL)	196.22	33.81	208.85	42.45	0.330
HDL-cholesterol (mg/dL)	60.02	13.74	55.32	18.17	0.390
LDL-cholesterol (mg/dL)	96.99	27.39	104.41	33.53	0.470
TG (mg/dL)	130.94	79.38	199.69	117.98	0.036
CRP (mg/L)	2.12	3.21	2.38	2.54	0.550
AST (U/L)	21.68	13.83	23.49	7.66	0.140
ALT (U/L)	7.53	6.66	9.95	5.50	0.025
Creatinine (µmol/L)	70.88	14.89	77.73	15.36	0.100
IL-6 (pg/mL)	1.23	1.59	1.78	2.25	0.450
Ferritin (ng/mL)	91.05	123.83	132.52	102.04	0.033
Homocysteine (µmol/L)	11.73	5.79	11.88	3.30	0.270
aTPO (IU/mL)	16.75	49.76	19.54	47.41	0.510

## Data Availability

The data supporting the findings of this study are available from the corresponding author (Ł.S.) upon request.

## Supplementary Materials

The following supporting information can be downloaded at: https://www.mdpi.com/article/10.3390/vaccines11010149/s1, Supplementary Table S1: Descriptive statistics: 3-week vs. 5-week interval group response in anti-S-ab concentrations during the 19-week observation period.
