# Supplementary material for: Immune Response to Vaccination against COVID-19 at Different Second-Dose Intervals and Their Associations with Metabolic Parameters
Source: Vaccines (Basel). 2023 Jan 10;11(1):149. doi: 10.3390/vaccines11010149 (PMC9865177; doi:10.3390/vaccines11010149)
Supplement: Supplementary file 1 [file vaccines-11-00149-s001.zip › vaccines-2098535-supplementary.pdf]

Group	Time after the 1st dose [weeks]	n	Arithmetic mean	Arithmetic SD	Geometric mean	Geometric SD	Geometric LOW CI95	Geometric UP CI95	Q1	Median	Q3	IQR
**3-week Interval**	0	54	0	0	0	0	0	0	0	0	0	0
**3-week Interval**	1	54	0,78375842	0,00590027	0	0	0	0	0	0	0	0
**3-week Interval**	2	54	31,6540741	44,0219502	0	0	0	0	6,9825	13,96	42,4125	35,43
**3-week Interval**	3	54	96,1360708	179,694745	47,3018955	3,84054934	33,0375516	67,7250342	32,855	58,28	97,7725	64,9175
**3-week Interval**	4	54	2501,87079	2112,46097	1670,27886	2,84509401	1263,77956	2207,53014	872,8	1915,5	3887,75	3014,95
**3-week Interval**	5	54	2600,69714	1816,05061	1916,53301	2,42660654	1512,96339	2427,75126	1040,75	2235,8821	3805,5	2764,75
**3-week Interval**	6	54	1997,4017	1425,07053	1486,24043	2,34600653	1183,89733	1865,79575	818,525	1816,5	2876	2057,475
**3-week Interval**	7	54	1703,56481	1183,39995	1289,02577	2,25875252	1037,23454	1601,93995	673,25	1450	2586,75	1913,5
**3-week Interval**	11	54	1438,57037	973,397813	1124,96187	2,13931844	918,429887	1377,93776	649,075	1281	2121,25	1472,175
**3-week Interval**	15	54	1363,00799	891,260642	1077,4133	2,12259472	881,45393	1316,93713	690,1	1238,5	1844	1153,9
**3-week Interval**	19	54	1191,31821	793,990043	934,070296	2,13716224	762,789269	1143,81174	602,575	1184	1514,70273	912,12773
**5-week Interval**	0	13	0	0	0	0	0	0	0	0	0	0
**5-week Interval**	1	13	0	0	0	0	0	0	0	0	0	0
**5-week Interval**	2	13	62,0838462	101,550524	0	0	0	0	0	11,49	80,14	80,14
**5-week Interval**	3	13	121,311078	152,537285	0	0	0	0	14,81	110,6	162,655031	147,845031
**5-week Interval**	4	13	116,866154	164,931943	37,0795699	5,64521325	14,4716015	95,0063824	14,14	24,74	181,1	166,96
**5-week Interval**	5	13	99,3630769	134,842672	38,2082981	4,72693491	16,4229826	88,8921383	11,6	31,86	130,5	118,9
**5-week Interval**	6	13	8337,78308	11268,6806	1975,2166	9,99023678	565,248142	6902,24407	961,4	1963	14322	13360,6
**5-week Interval**	7	13	10544,2403	9553,38945	6762,2033	2,97437994	3738,92699	12230,0846	4740	6832	11510	6770
**5-week Interval**	11	13	4779,4877	4352,50991	3173,18398	2,77051869	1823,54425	5521,71769	2451	3731	4737	2286
**5-week Interval**	15	13	3494,41144	2659,76317	2576,3676	2,4315564	1589,42013	4176,15827	2003	2505	4655,64304	2652,64304
**5-week Interval**	19	13	1579,93691	1062,82864	1203,69701	2,35546184	755,534542	1917,69722	1110	1479	1892,46222	782,462224

Supplementary Table S1. Descriptive statistics: 3-week vs. 5-week interval group response in Anti-S-ab concentrations in 19-week observation.
